# Supplementary figures and images for: Carbon system state determines warming potential of emissions
Source: PLoS One. 2024 Aug 1;19(8):e0306128. doi: 10.1371/journal.pone.0306128 (PMC11293723; doi:10.1371/journal.pone.0306128)

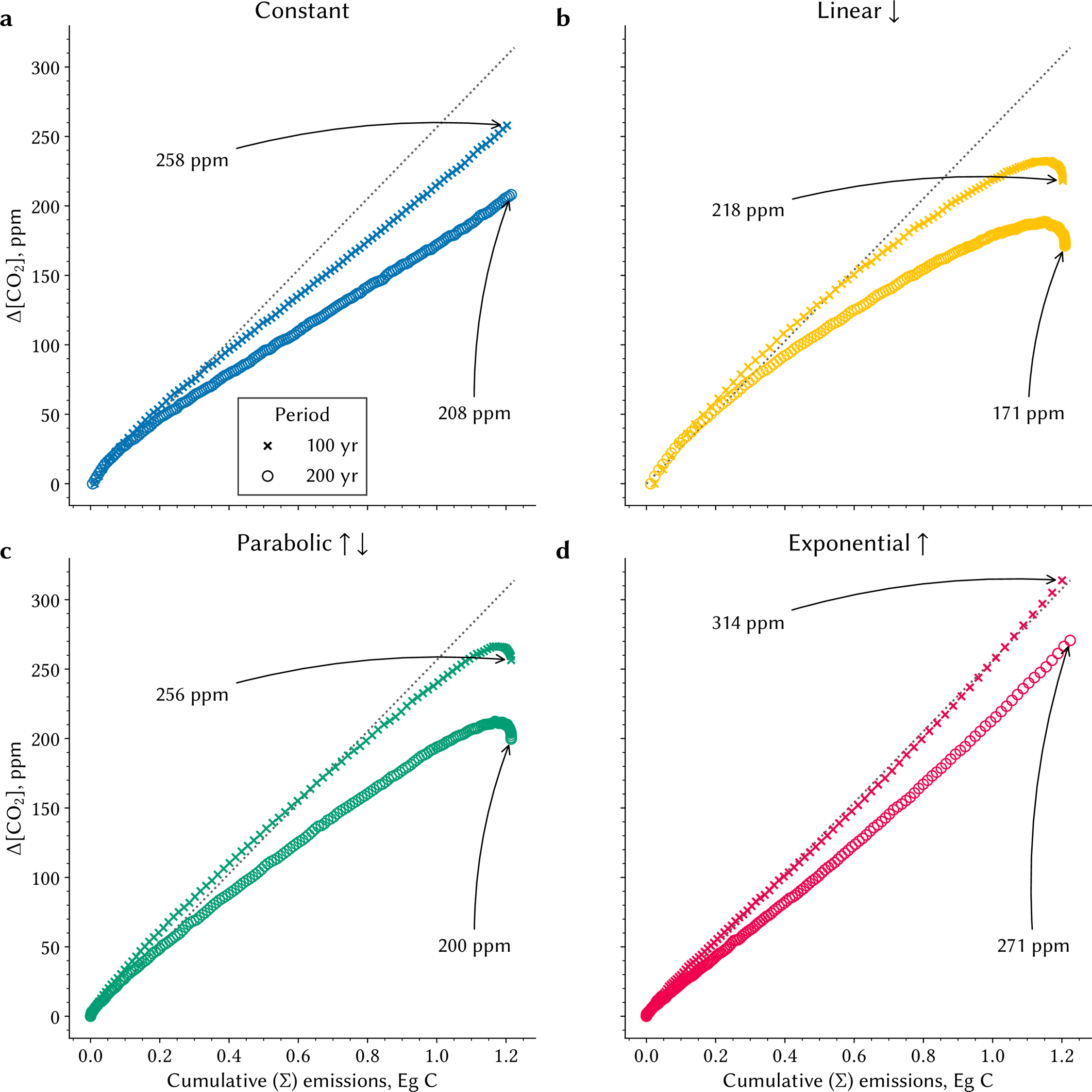

Supplement: S1 Fig — a–d Relationship between cumulative carbon emissions (x-axis) and the change in atmospheric CO2 concentration (Δ[CO2]) is shown, where the colors refer to the four different pathways analogous to Fig 1 and the markers refer to the different time periods. The arrows annotate the final Δ[CO2] in each model experiment. The dotted black line facilitates comparison between the pathways. (TIF) [file pone.0306128.s001.tif]

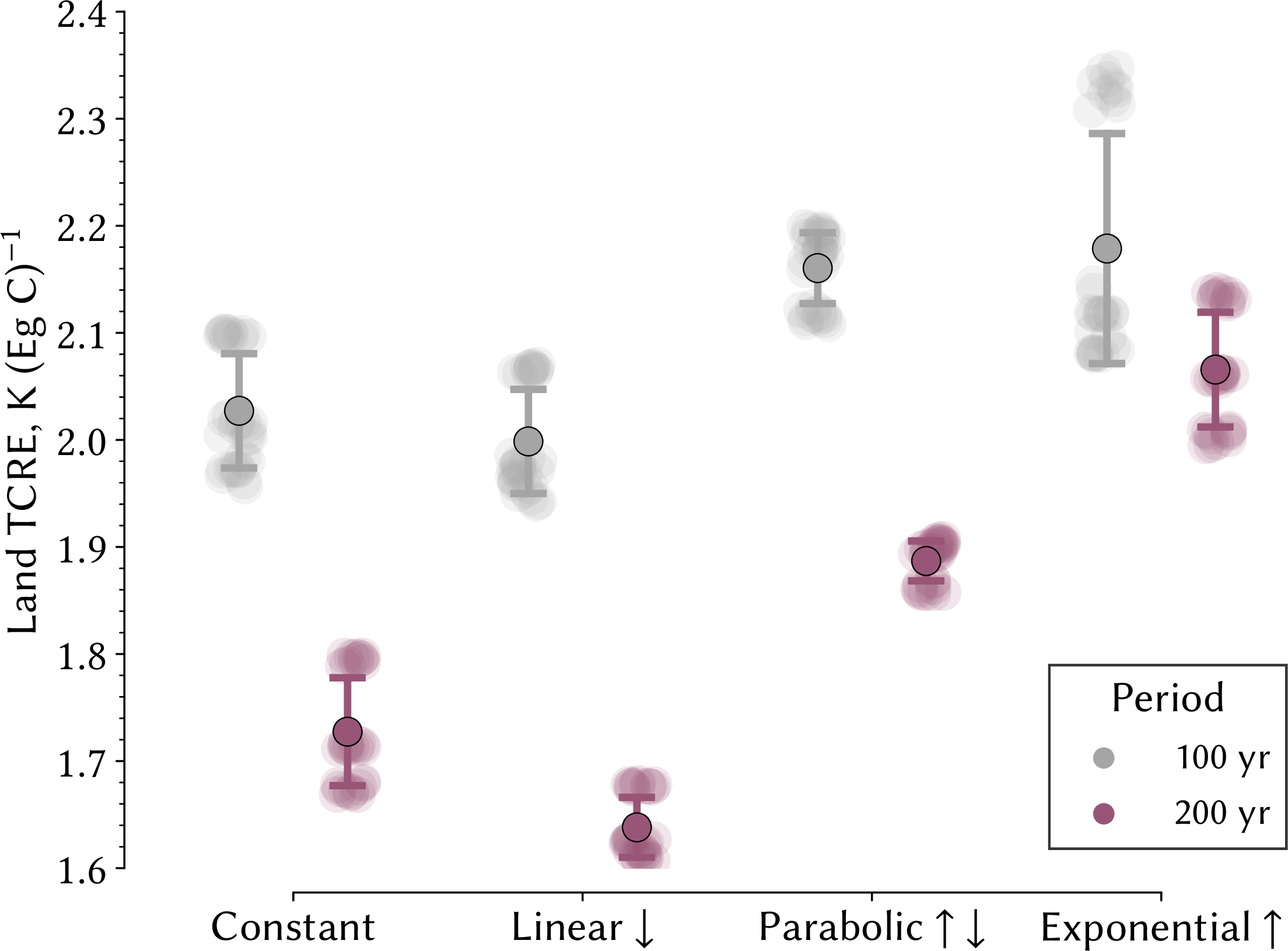

Supplement: S2 Fig — Transient Climate Response to Cumulative Emissions (TCRE) for different pathways (x-axis) and simulation periods (colors), focusing on the warming of the land surface. Land TCRE is estimated using the conventionally used linear regression method [13]. Shaded dots exhibit the spread in the estimates of the final five years when 1.2 Eg C have been emitted as well as among different realizations. Pointplot with whiskers show the mean and standard deviation of the spread. (TIF) [file pone.0306128.s002.tif]

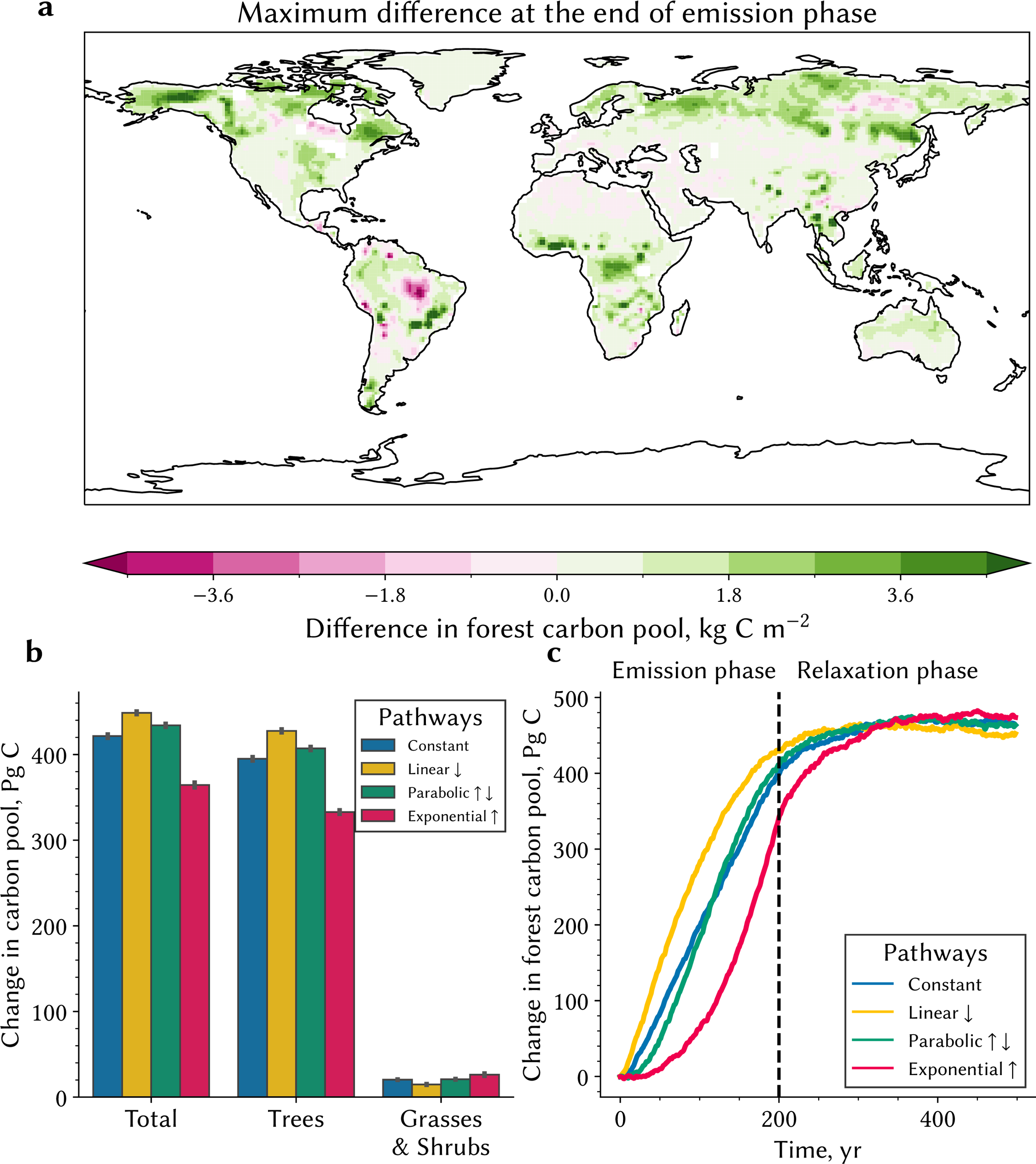

Supplement: S3 Fig — a Global map of the difference in the total forest carbon pool between the L and E pathways (yellow and red in b and c, respectively) at the end of the emission phase, calculated with MPI-ESM1–2-LR in the 200-year runs. b Change in carbon pool for forest, grass- and shrublands, and total vegetation for the different emission pathways. The whiskers represent the uncertainty between the different realizations. c Change of forest carbon pool across the four pathways as a function of time for both the emission and relaxation phases. (TIF) [file pone.0306128.s003.tif]

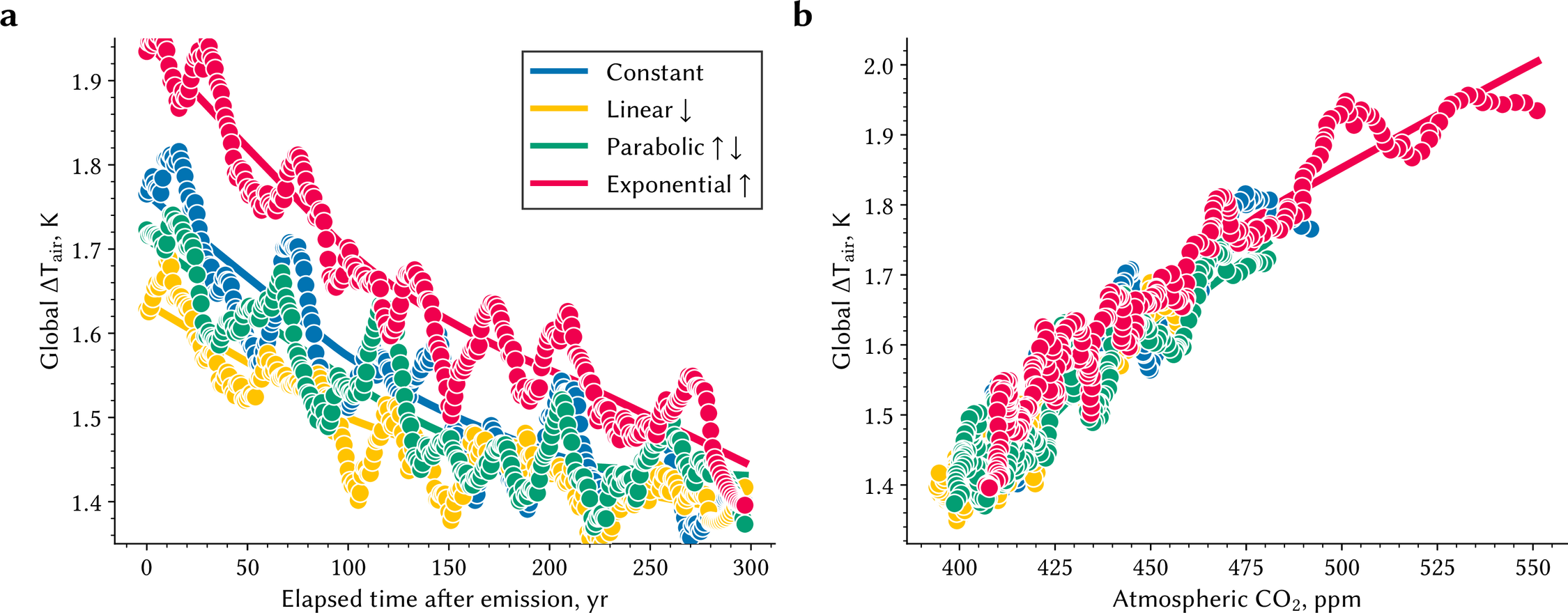

Supplement: S4 Fig — a Different colored dots depict the different emission pathways smoothed by a low-pass filter (Savitzky-Golay filter), where the lines represent a nonparametric LOWESS fit. b as in a, but with atmospheric CO2 as x-axis. (TIF) [file pone.0306128.s004.tif]

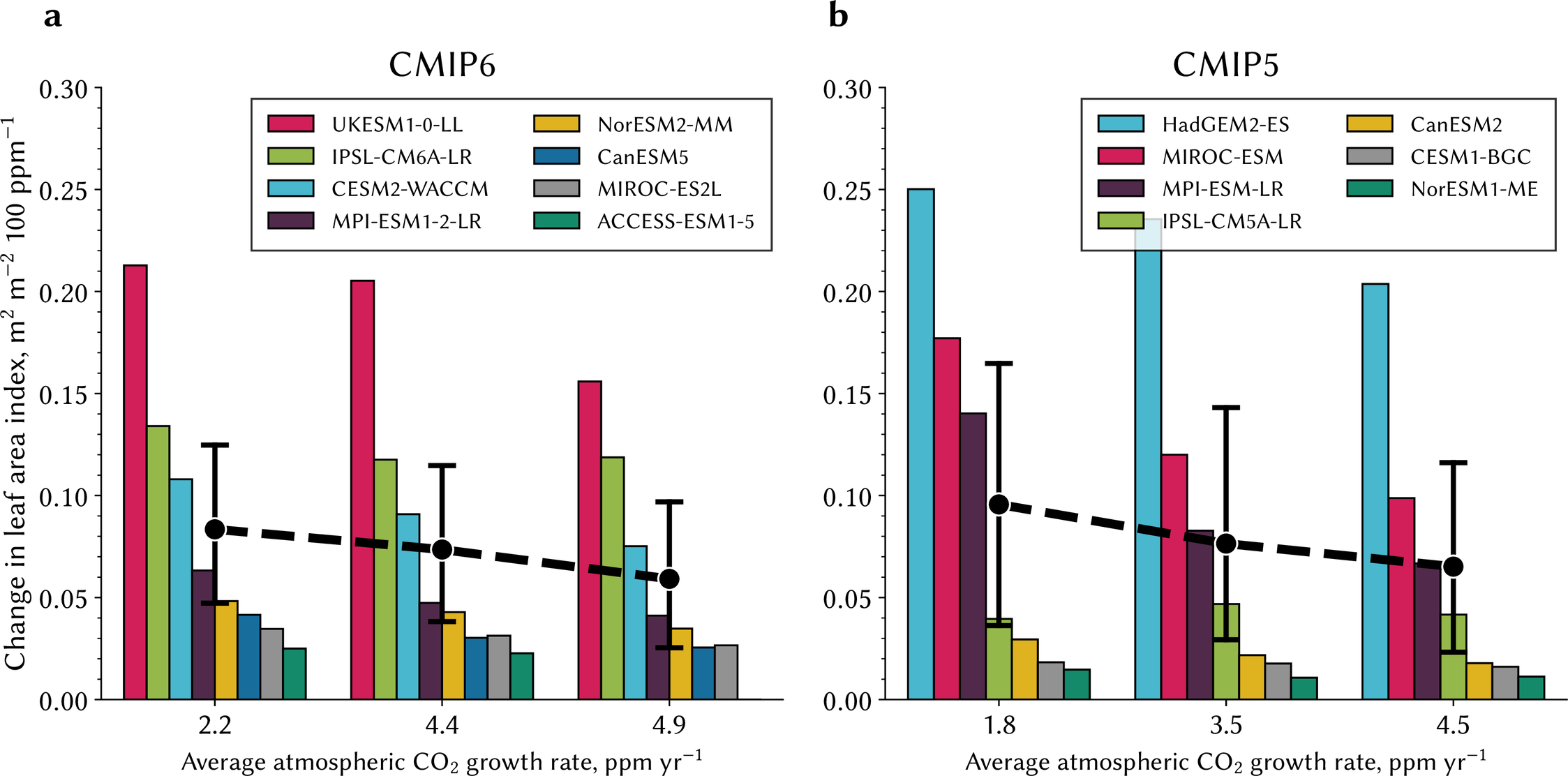

Supplement: S5 Fig — a Change in leaf area index at latitudes above 60° N per a 100 ppm change in atmospheric CO2 across three different future scenarios in CMIP6 reflecting different CO2 growth rates (x-axis). The black dot refers to the multi-model mean value for each growth rate, where the whiskers represent the standard deviation. b as in c but for CMIP5. (TIF) [file pone.0306128.s005.tif]

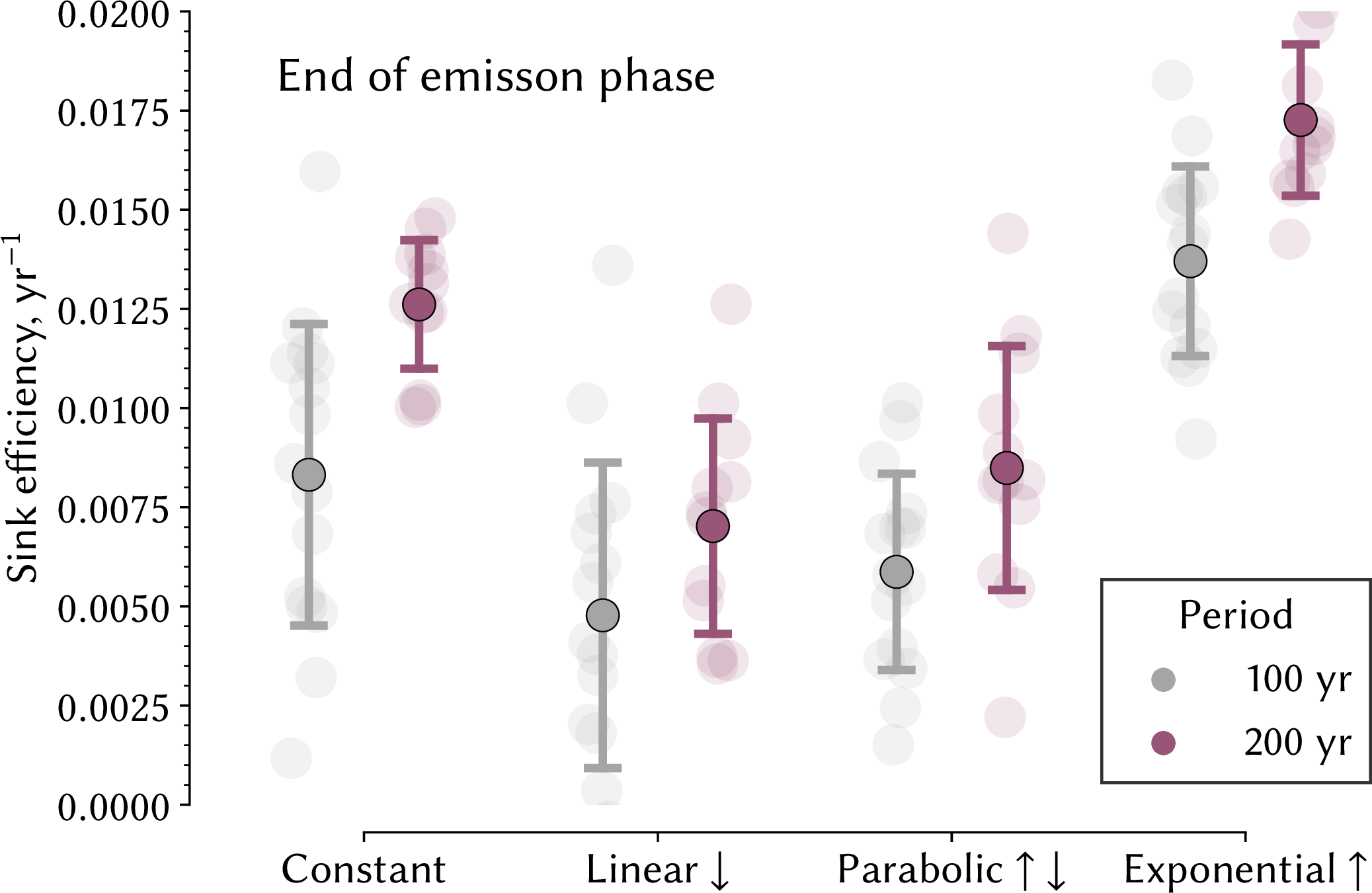

Supplement: S6 Fig — Sink efficiency for different pathways (x-axis) and simulation periods (colors) at the end of the emission phase. Shaded dots exhibit the spread in the estimates of the final five years when 1.2 Eg C have been emitted as well as among different realizations. Pointplot with whiskers show the mean and standard deviation of the spread. (TIF) [file pone.0306128.s006.tif]

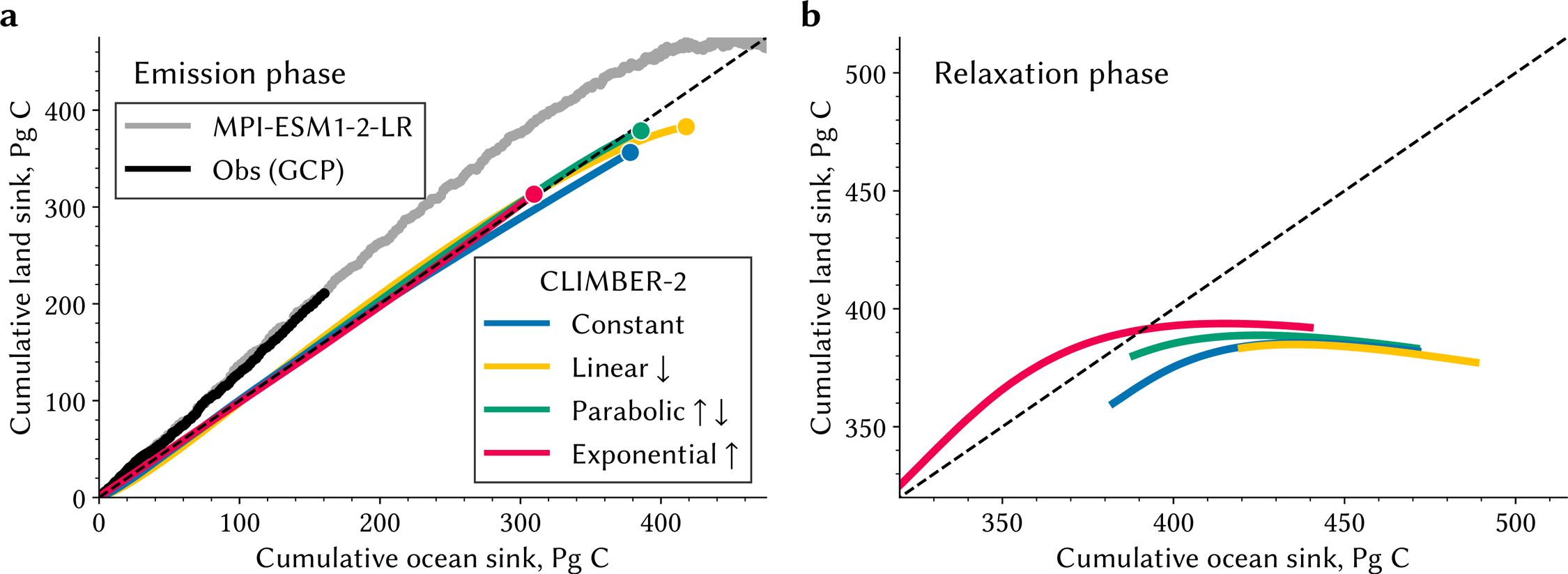

Supplement: S7 Fig — a Cumulative land and ocean sinks are juxtaposed across all pathways. The markers denote the final state at the end of the emission phase of 1.2 Eg C. The dashed line indicates equal magnitude of cumulative land and ocean sinks. The gray and black lines refer to the relationships inferred from MPI-ESM1.2-LR simulations and GCP estimates, respectively. b As in a, but for relaxation phase. The Climate-Biosphere model (version 2) CLIMBER-2 is an Earth System Model of Intermediate Complexity (EMIC) and consists of a statistical-dynamical atmosphere component (51° × 10° spatial resolution), a 2D ocean component with three zonally averaged basins, and a land component including dynamic vegetation [49]. The same model version of CLIMBER-2 is used, which was also used for the ZecMIP runs [6]. (TIF) [file pone.0306128.s007.tif]

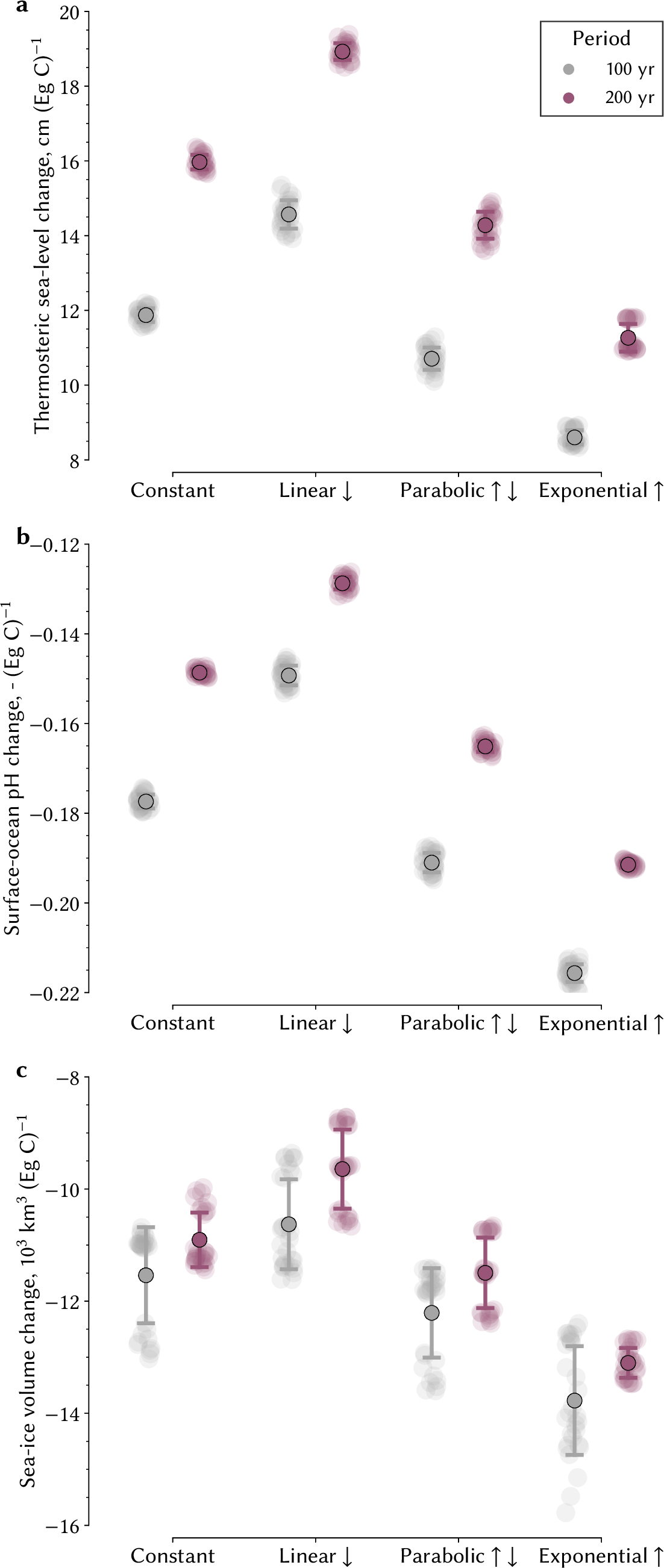

Supplement: S8 Fig — a. Thermosteric sea-level change for given emission of 1 Eg C for different pathways estimated using a linear regression method analogous to TCRE in Fig 1d; [13]. Shaded dots exhibit the spread in the estimates of the final five years when 1.2 Eg C have been emitted as well as among different realizations. Pointplot with whiskers show the mean and standard deviation of the spread. b as in a but for surface-ocean pH change. c as in a but for sea-ice volume change. (TIF) [file pone.0306128.s008.tif]
