# Supplementary material for: Carbon system state determines warming potential of emissions
Source: PLoS One. 2024 Aug 1;19(8):e0306128. doi: 10.1371/journal.pone.0306128 (PMC11293723; doi:10.1371/journal.pone.0306128)
Supplement: S1 Table — (PDF) [file pone.0306128.s009.pdf]

Best-fit values for parameters of the double exponential decay function (see Equation 14)

Model	η	τ_F	τ_S	C_T^0	C_{OL}^0	R^2
Unit	-	yr	yr	Pg C	Pg C	-
**ZecMIP (1000 Pg C)**						
ACCESS-ESM1-5	0.13 ± 0.008	20.29 ± 2.01	512.15 ± 29.48	586.78 ± 1.69	418.37	0.996
CESM2	0.34 ± 0.002	28.01 ± 0.22	807.37 ± 12.78	579.10 ± 0.37	425.60	1.000
CanESM5	0.33 ± 0.002	21.74± 0.30	1033.38± 14.78	498.36 ± 1.06	498.02	0.998
GFDL-ESM4	0.31 ± 0.003	26.12 ± 0.58	598.97 ± 11.19	505.69 ± 1.18	496.66	0.998
MIROC-ES2L	0.22 ± 0.003	23.88 ± 0.71	660.19 ± 8.10	516.92 ± 1.49	472.20	0.997
MPI-ESM1-2-LR	0.25 ± 0.003	21.87 ± 0.52	383.24 ± 4.08	536.14 ± 1.17	464.27	0.999
NorESM2-LM	0.35 ± 0.005	27.62 ± 0.42	639.66 ± 28.25	564.94 ± 0.41	439.26	1.000
UKESM1-0-LL	0.17 ± 0.002	30.98 ± 0.76	808.04 ± 7.59	568.75 ± 0.92	426.34	0.998
**MPI-ESM1-2-LR Pathways (1200 Pg C)**						
Constant	0.28± 0.01	70.92 ± 2.58	1152.43 ± 54.47	434.98 ± 0.72	773.10	0.998
Linear ↓	0.24 ± 0.01	80.40 ± 3.96	1694.86 ± 127.91	364.70 ± 0.59	843.32	0.997
Parabolic ↑ ↓	0.31 ± 0.01	92.89 ± 4.05	1700.77 ± 163.26	417.87 ± 0.58	793.13	0.998
Exponential ↑	0.35 ± 0.01	61.72 ± 1.67	943.89 ± 36.29	559.11 ± 1.04	652.48	0.998

Estimates including the standard error (68% confidence) are shown for the CMIP6 ZecMIP multi-model ensemble and the four different emission pathways simulated using MPI-ESM1-2-LR.
