# Supplementary material for: Carbon system state determines warming potential of emissions
Source: PLoS One. 2024 Aug 1;19(8):e0306128. doi: 10.1371/journal.pone.0306128 (PMC11293723; doi:10.1371/journal.pone.0306128)
Supplement: S2 Table — (PDF) [file pone.0306128.s010.pdf]

Best-fit values for parameters of the double exponential decay function (Equation 14), where η is sampled from the parameter space determined in Table S1.

Model	η	τ_F	τ_S	C_T^0	C_{OL}^0	R^2
Unit	-	yr	yr	Pg C	Pg C	-
**ZecMIP (1000 Pg C)**						
ACCESS-ESM1-5	[0.13 – 0.35]	20.52 ± 3.66	519.06 ± 63.81	586.29 ± 0.82	418.37	0.995 ± 0.000
CESM2	[0.13 – 0.35]	28.40 ± 5.39	1037.60 ± 622.43	577.74 ± 2.53	425.60	0.999 ± 0.001
CanESM5	[0.13 – 0.35]	23.33 ± 3.52	1269.50 ± 628.47	493.79 ± 11.17	498.02	0.992 ± 0.005
GFDL-ESM4	[0.13 – 0.35]	27.47 ± 5.50	647.11 ± 168.05	502.13 ± 3.07	496.66	0.997 ± 0.001
MIROC-ES2L	[0.13 – 0.35]	26.22 ± 5.03	689.80 ± 95.04	512.74 ± 5.50	472.20	0.995 ± 0.001
MPI-ESM1-2-LR	[0.13 – 0.35]	23.17 ± 4.37	394.77 ± 50.94	532.64 ± 3.23	464.27	0.998 ± 0.001
NorESM2-LM	[0.13 – 0.35]	27.57 ± 4.25	830.64 ± 521.07	564.98 ± 2.09	439.26	1.000 ± 0.000
UKESM1-0-LL	[0.13 – 0.35]	33.26 ± 6.19	831.35 ± 92.55	565.85 ± 3.03	426.34	0.998 ± 0.001
**MPI-ESM1-2-LR Pathways (1200 Pg C)**						
Constant	[0.24 – 0.35]	75.53 ± 10.00	1318.27 ± 291.27	434.38 ± 1.27	773.10	0.998 ± 0.000
Linear ↓	[0.24 – 0.35]	101.18 ± 11.81	3483.91 ± 1738.02	363.31 ± 0.74	843.32	0.997 ± 0.000
Parabolic ↑ ↓	[0.24 – 0.35]	90.62 ± 10.56	1708.37 ± 438.07	418.08 ± 0.84	793.13	0.998 ± 0.000
Exponential ↑	[0.24 – 0.35]	50.18 ± 7.10	742.98 ± 111.07	561.10 ± 0.97	652.48	0.997 ± 0.001

The uncertainty estimate refers to one standard deviation of the individual parameter estimates between the samples. Results are shown for the CMIP6 ZecMIP multi-model ensemble and the four different emission pathways simulated using MPI-ESM1-2-LR.
