# Supplementary material for: Carbon system state determines warming potential of emissions
Source: PLoS One. 2024 Aug 1;19(8):e0306128. doi: 10.1371/journal.pone.0306128 (PMC11293723; doi:10.1371/journal.pone.0306128)
Supplement: Attachment — Submitted filename: first-round-review.pdf [file pone.0306128.s011.pdf]

General comments

Winkler et al. provide an analysis of the warming caused per unit of CO2 emissions (the TCRE) using a state-of-the-art earth system model. They use a series of idealised experiments to analyse the path-/state-dependence of the TCRE. They conclude that it is the carbon system state which determines TCRE, rather than the TCRE being state-/path-independent, the most standard assumption currently made in the literature.

The paper is well-written and clear, for which I thank the authors. I think the model experiments are also clearly run and can't see any immediate errors. However, I have a number of major concerns which means I cannot recommend acceptance of this manuscript in its current form. These mainly relate to the justification of the key claim and the logic the authors follow to reach their key conclusion. Other concerns relate to acknowledgement of other literature, framing and reproducibility. I outline these concerns, but encourage the authors to re-submit as they bring a very nice presentation style to their results and it would be great to see this story clarified and published.

Major concerns

Justification of key claim

The key claim of the paper seems to be that the TCRE is not constant, but it is well predicted by the state of the carbon cycle. It is very hard to assess this claim, as the key figure needed is not in the paper. The key figure required is S2, with all pathways on the same axis (which can also be thought of as being the same as Figure 1c, with the y-axis being temperature rather than atmospheric CO2). I was very surprised that such a figure was not included and, eyeballing the figure, it looks to me like the TCRE would be very similar across the pathways.

I am more convinced by Figure 4a and it's accompanying analysis that the sink apportionment is path-independent. However, it would be important to see the same figure produced with other models to see if this is a model-specific effect. Such a figure would be important to justify the (currently unjustified) statement on page 10-11, line 226-228: "The effect of the state only becomes apparent when considering both a more comprehensive representation of the terrestrial and oceanic carbon cycle in a fully coupled carbon-climate system." To prove this, the authors would need to show that other models fail to capture this and explain why it only appears in the more fully coupled representations, and such analysis is currently lacking. Without this analysis, this claim should be re-considered or caveated.

However, I am not convinced by the claims that the TCRE is so path-dependent and I currently can't tell how the authors are coming to their conclusion about the issues with the TCRE. I am guessing that this is somehow related to the TCRE calculation, which is done on the last 5 years of the simulation rather than

over the entire simulation (which might explain why there are relatively larger variances in it than I would expect from a regression over the entire experiment), but I currently can't be sure (see also issues with reproducibility which make it hard for me to tell exactly what has been done).

Further on this point, I am not convinced that the idealised pathways shown are actually suitable for fully justifying the current conclusions of the paper. The spread in mitigation scenarios is much narrower than the spread between the 4 idealised pathways shown here. Realistically, we are not choosing between constant, parabolic, linear down and exponential. Rather, we are looking at variations of parabolic depending on future mitigation action. To really identify the extent to which the TCRE is scenario-dependent in mitigation scenarios, I think further experiments are required which examine the TCRE over a range of scenarios which are more similar to each other and so cover the possible future space with greater density. Without such simulations, I think the paper is still publishable, but its conclusions need to be caveated more heavily.

Finally on this point, I wonder if other simulations could more clearly prove the point the authors are making about the importance of the carbon state. For example, could the authors spin the carbon cycle up to some state in different ways, then apply the same emissions thereafter. If their hypothesis is correct, the fact that the carbon cycle followed different paths to get to the same state shouldn't matter for future temperature evolution. Some experiments that are more targeted at this state-independence would improve the strength of their conclusions I feel. As above, without such simulations, I think the paper is still publishable, but its conclusions need to be caveated more heavily.

Logic around what is expected and what is surprising

The paper seems to misrepresent (or its authors misunderstand) what is expected and what is not in terms of the relationship between cumulative CO2 emissions, atmospheric CO2 and warming. For example, the difference between pathways in Figure 1c is exactly what you expect. The relationship between cumulative CO2 emissions and atmospheric CO2 is pathway-dependent. Similarly, the relationship between atmospheric CO2 and warming is pathway-dependent. It is the cancellation between these two pathway-dependent relationships that gives rise to the approximately pathway-independent relationship between cumulative CO2 emissions and warming. If Figure 1c showed warming against cumulative CO2 emissions then this would be a more unexpected result (but even then, the difference pathways would not be so big relative to overall uncertainty, see also comments about framing below).

Given this context, there are a few statements which need to be fixed.

page 6, line 126-127: "The variation in atmospheric carbon at the end of the emission phase is larger across pathways than models (200 vs. 100 Pg C), which is contrary to expectation". As above, this is exactly what you would expect. If the statement were about the variation in warming then it would be different,

but this statement is about the variation in atmospheric CO2 so this variation across pathways is completely in line with expectations.

page 6, line 148-150: "Thus, the emergent decay rates are not a model-specific property but depend on the forcing, contrary to previous reporting". I think this understanding of the state-dependence of decay rates is well understood in the literature, indeed it is this state-dependence which motivated the entire design of the FaIR model (Millar et al., 2017). Once again, I don't think this characterisation of previous reporting or differences from current understanding is correct.

page 8, line 167-168: "This time dependency in the terrestrial carbon cycle, hitherto unacknowledged, serves to highlight the dependency of the system on forcing timeline." As above, if this were a discussion of warming then the statement would be correct. As it relates to the state of the carbon cycle, I don't think the statement nor its implications about the state of the literature ("hitherto unacknowledged") are correct.

Framing

The paper is written in such a way that, at least to my reading, suggests we need to completely re-think the use of the remaining carbon budget concept. For example, the statement, "Therefore, the emphasis on remaining carbon budget to limit surface warming in accordance with the Paris Agreement requires a critical examination". I don't think such a framing is justifiable.

The remaining carbon budget is a key concept in climate science that has been heavily examined for over a decade. It is probably true that its use often needs to be more clearly accompanied with the caveat that it is a first-order approximation, and that more sophisticated tools are required for more precise estimates. However, using a linear TCRE is often still appropriate because of the wide uncertainties associated with the TCRE. For example, in the latest IPCC report the likely (17-83%) range for the TCRE was 1.0 - 2.3 K / TtC. Against an uncertainty of this size, a +/- 20% deviation away from a constant TCRE is irrelevant for most use cases, such as for the IPCC's reporting of remaining carbon budgets. The authors must do a better job of capturing the size of the deviation they are discussing compared to wider uncertainties in the TCRE if they are to frame this paper appropriately and to highlight the circumstances in which their analysis requires a re-think and the circumstances in which the corrections they are discussing are small compared to other uncertainties.

Precision

Related to the above point, it would be easier for the reader to understand the size of the deviations being discussed if the authors removed all vague quantifications, such as "roughly constant" in the abstract. A similarly vague quantification is used on page 10, line 206-207: "However, at the same state of the system, the sink efficiency is the same irrespective of the pathway property (Figure 4c)." In

the figure, all the dots are clearly not the same so it would be easier if these statements were quantified, rather than only described qualitatively. This would also remove the need for the authors to judge what is the same and what is different (a choice I can't really understand as the dots in Figure 4c seem to be as spread as in other figures yet they are described as the same whereas a similar spread is described as a difference elsewhere).

Minor concerns

Acknowledgement of other studies

The authors frame this paper in a way that suggests that everyone is assuming the TCRE is perfectly linear in all cases and no-one has ever considered otherwise. For example, the sentence ending in, "which heretofore have been mainly overlooked", in the abstract. I don't think this is a fair framing.

There have been studies which consider non-linearities in the TCRE and their impact (see, e.g. Nicholls et al., 2020; Avakumović. 2023 and references therein). These studies should be acknowledged and considered. (I assume the authors will disagree with the models that sit underneath these studies, but that is besides the point, which is that people have thought about path- and state-dependency in the TCRE already and presenting the literature as if no-one has is misleading.)

Reproducibility

As a reviewer, part of my job is to check the reproducibility of the results. I acknowledge that this is rarely done in climate science for a whole range of reasons (hence why this is a minor concern rather than major), but I think it is worth trying to do this better here. My key concerns are that, as a reviewer, I cannot:

- inspect or run any of the code used for downloading and processing the CMIP results
- download or run any of the processing/plotting code (if I could, maybe my complaint about missing figures could have been dealt with by me just making the figure myself)
- access any of the model output required to make the figures/reproduce the analysis without asking the author
    - I strongly encourage the authors to find a way to make the output data publicly available, there are solutions for dealing with large data files so I don't think that is a fair justification for not releasing the data (and if concerns about being scooped are part of the reason, a suitable license for data access should deal with this)
- inspect any of the scripts used to configure and run MPI-ESM. Having read through MPI-ESM's RECIPE file, this is clearly an involved process. In an ideal world, the ability to re-run the model would be there. However, given MPI-ESM's run setup is specific to MPI's computing environment,

that doesn't seem to be possible (which is the standard in climate science unfortunately). Given this, at a minimum the run scripts and any custom inputs should be archived next to this paper to allow for at least a non-zero chance of reproducibility.

Figures

Very nice and clear, thank you. One small question:

- Is it possible to add uncertainty bars/shading onto the GCP estimate in Fig S7 and Fig 4a?

Technical corrections

page 3, line 29: 'time sequence and duration' → 'timing'

page 3, line 31: delete 'likely' (unless there is some justification, in which case add it)

page 5, line 92: 'into the' → 'into'

page 7, line 156-157: "These model intercomparison exercises lack dynamics arising from an interactive carbon cycle as these are CO2 concentration driven simulations only" That's only true if you don't have a cumulative CO2 emissions axis in your analysis/plots. If you have that axis, there has to be some carbon cycle influence in there no (because the carbon cycle determines uptake, so you will get variations even though it is a concentration-driven experiment)?

page 15, line 294: "negligible" → "negligibly"

page 16, line 317-318: "resulting in strong vertical gradients in the mixing ratio of CO2, causing the model to crash", sounds painful, well done for working out how to handle that

page 31, line 602-603: reference incomplete, current text says "point to methods"

page 32, line 618: 'but for' → 'but for the'

References

Avakumović, V.: Carbon budget Concept, its Deviation and the Equation: Climate Economics Perspective, Earth Syst. Dynam. Discuss. [preprint], https://doi.org/10.5194/esd-2023-7, in review, 2023.

Millar, R. J., Nicholls, Z. R., Friedlingstein, P., and Allen, M. R.: A modified impulse-response representation of the global near-surface air temperature and atmospheric concentration response to carbon dioxide emissions, Atmos. Chem. Phys., 17, 7213–7228, https://doi.org/10.5194/acp-17-7213-2017, 2017.

Nicholls et al. (2020): Z R J Nicholls et al 2020, Implications of non-linearities between cumulative CO2 emissions and CO2-induced warming

for assessing the remaining carbon budget, Environ. Res. Lett. 15 074017. https://iopscience.iop.org/article/10.1088/1748-9326/ab83af
