# Supplementary material for: Carbon system state determines warming potential of emissions
Source: PLoS One. 2024 Aug 1;19(8):e0306128. doi: 10.1371/journal.pone.0306128 (PMC11293723; doi:10.1371/journal.pone.0306128)
Supplement: Attachment — Submitted filename: response-to-ref-02.pdf [file pone.0306128.s012.pdf]

Authors' Response to Referee 2 (PONE-D-23-32708)

April 1, 2024

Winkler et al. provide an analysis of the warming caused per unit of CO_2 emissions (the TCRE) using a state-of-the-art earth system model. They use a series of idealised experiments to analyse the path-/state-dependence of the TCRE. They conclude that it is the carbon system state which determines TCRE, rather than the TCRE being state-/path-independent, the most standard assumption currently made in the literature. The paper is well-written and clear, for which I thank the authors. I think the model experiments are also clearly run and can't see any immediate errors. However, I have a number of major concerns which means I cannot recommend acceptance of this manuscript in its current form. These mainly relate to the justification of the key claim and the logic the authors follow to reach their key conclusion. Other concerns relate to acknowledgement of other literature, framing and reproducibility. I outline these concerns, but encourage the authors to re-submit as they bring a very nice presentation style to their results and it would be great to see this story clarified and published.

We thank the reviewer for the detailed and critical assessment of our manuscript and the acknowledgment of its relevance and encouragement for publication. In the following we address each comment, including the changes in the revised manuscript. Reviewer comments are typeset in italic blue font and our responses in black font.

1 Major Concerns

1.1 ***Justification of key claim*** *The key claim of the paper seems to be that the TCRE is not constant, but it is well predicted by the state of the carbon cycle. It is very hard to assess this claim, as the key figure needed is not in the paper. The key figure required is S2, with all pathways on the same axis (which can also be thought of as being the same as Figure 1c, with the y-axis being temperature rather than atmospheric CO_2). I was very surprised that such a figure was not included and, eyeballing the figure, it looks to me like the TCRE would be very similar across the pathways.*

Yes, the illustrated pathway dependence is not a game changer in terms of TCRE and defining carbon budgets, but there are notable and non-negligible differences (see range from 1.63 K to 1.94 K in Figures S2; and Table 1). We further include an in-depth decomposition of TCRE that illustrates the pathway dependence in the system (Figure R2-1). Furthermore, we toned down the key claim in various locations in the revised manuscript — clearly illustrated in the resubmitted manuscript with highlighted changes.

1.2 *I am more convinced by Figure 4a and it's accompanying analysis that the sink apportionment is path-independent. However, it would be important to see the same figure produced with other models to see if this is a model-specific effect. Such a figure would be important to justify the (currently unjustified) statement on page 10-11, line 226-228: "The effect of the state only becomes apparent when considering both a more comprehensive representation of the terrestrial and oceanic carbon cycle in a fully coupled carbon-climate system." To prove this, the authors would need to show that other models fail to capture this and explain why it only appears in the more fully coupled representations, and such analysis is currently lacking. Without this analysis, this claim should be re-considered or caveated.*

We thank the reviewer for pointing out the concern. We only had access and resources to run the experiments with one other model, CLIMBER-2 (Figure 8 in revised manuscript). These model runs confirm the findings, but further models should repeat such experiments in the described setting. However, only a few models consider the paramount time-scales of the terrestrial carbon

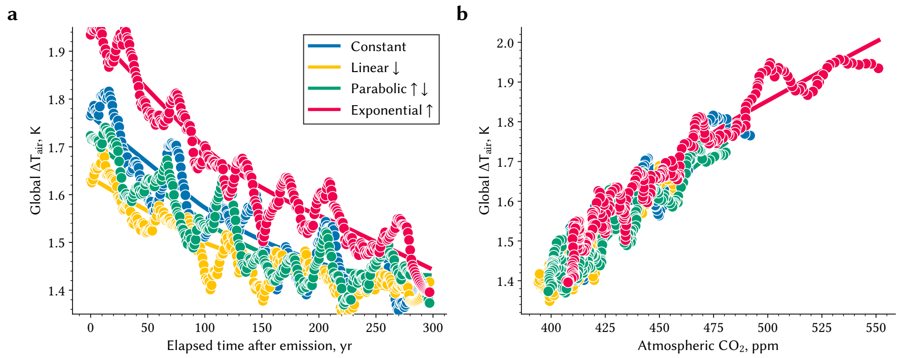

Figure R2- 1: **Evolution of global mean surface air temperature across pathways after emission stop.** **a** Different colored dots depict the different emission pathways smoothed by a low-pass filter (Savitzky-Golay filter), where the lines represent a nonparametric LOWESS fit. **b** as in **a**, but with atmospheric CO_2 as x-axis.

cycle and are suitable to investigate these dynamics. Also, we rephrased the sentence pointed out by the reviewer to reflect a more justifiable claim:

"*Our model experiments suggest that the effect of the state becomes apparent when considering both a more comprehensive representation of the terrestrial and oceanic carbon cycle in a fully coupled carbon-climate system.*"

1.3 *However, I am not convinced by the claims that the TCRE is so path-dependent and I currently can't tell how the authors are coming to their conclusion about the issues with the TCRE. I am guessing that this is somehow related to the TCRE calculation, which is done on the last 5 years of the simulation rather than over the entire simulation (which might explain why there are relatively larger variances in it than I would expect from a regression over the entire experiment), but I currently can't be sure (see also issues with reproducibility which make it hard for me to tell exactly what has been done).*

We agree with the reviewer that the section on how we conduct the linear regression to estimate TCRE is not described in enough detail. We follow the approach used by Williams et al. (2020) for all CMIP5 and CMIP6 models. Accordingly, TCRE is diagnosed from the least-squares regression slope $\Delta T(t)$ against cumulative emissions $\Sigma\, E(t)$, where the regression is calculated from the start of the time series to year t. For instance, TCRE for year 50 in each simulation is based on the regression over the first 50 years, while TCRE for year 200 is based on the regression over the entire time-series of 200 years. We discard the first 10 years of each simulation as it takes a few years until the signal emerges from internal variability. For each experiment we report the mean TCRE value and standard deviation over the three ensemble members and the final five years before reaching 1200 PgC total emissions in each simulations. We do not force the regression algorithm to go through zero. The estimated intercept is close to zero. We use two other methods to identify changes in temperature and we included the following section for more details on that matter:

"*We derive and decompose TCRE primarily following the methodology detailed in Williams et al. (2020). Throughout this study, TCRE is computed using three methods: the expanding-window regression, delta, and a low-pass filter method. In the expanding-window regression method, TCRE is estimated using the slope in regressing the change in surface temperature against cumulative carbon emissions. The regression is computed iteratively in an expanding-window approach to monitor the temporal evolution and the stability of the TCRE estimate, excluding the initial ten years due to high natural variability. We report the average and standard deviation for the last ten regression estimates, i.e., estimates close to full emission, and for all three realizations (Figure 1). The delta method calculates the change in surface warming relative to pre-industrial equilibrium, reporting mean and standard deviation over all three realization and final five years. For TCRE calculation, these values can be divided by the total carbon emissions of 1.2 Eg C, while plain temperature deltas are included*

in Table 1 for comparability with other estimates based on the 2 K carbon budget. In the low-pass filter method, we smooth year-to-year variability using a Savitzky-Golay filter and average the results across the three realizations for each experiment, as shown in Figure 2a."

1.4 *Further on this point, I am not convinced that the idealised pathways shown are actually suitable for fully justifying the current conclusions of the paper. The spread in mitigation scenarios is much narrower than the spread between the 4 idealised pathways shown here. Realistically, we are not choosing between constant, parabolic, linear down and exponential. Rather, we are looking at variations of parabolic depending on future mitigation action. To really identify the extent to which the TCRE is scenario-dependent in mitigation scenarios, I think further experiments are required which examine the TCRE over a range of scenarios which are more similar to each other and so cover the possible future space with greater density. Without such simulations, I think the paper is still publishable, but its conclusions need to be caveated more heavily. Finally on this point, I wonder if other simulations could more clearly prove the point the authors are making about the importance of the carbon state. For example, could the authors spin the carbon cycle up to some state in different ways, then apply the same emissions thereafter. If their hypothesis is correct, the fact that the carbon cycle followed different paths to get to the same state shouldn't matter for future temperature evolution. Some experiments that are more targeted at this state-independence would improve the strength of their conclusions I feel. As above, without such simulations, I think the paper is still publishable, but its conclusions need to be caveated more heavily.*

We acknowledge the reviewer's suggestion regarding the potential benefits of conducting additional simulations to thoroughly test the hypothesis. Our primary aim was to utilize idealized simulations to explore the dynamics of the system in a more generalized manner, rather than focusing on specific scenarios with predefined assumptions about 21st-century development. Unfortunately, conducting further simulations, particularly spin-ups to different equilibria of the carbon-cycle climate system within a fully coupled Earth system model (ESM), presents challenges and significant costs. We followed the reviewer's recommendation and rewrote the conclusions to focus on unaccounted uncertainty in TCRE linked to these non-linearities.:

"*While these non-linearities do not entirely invalidate TCRE, they do introduce additional uncertainty. If the model-specific range of 0.25 °C for TCRE, as quantified in this study, were to apply in all models in this fully-coupled setting, it could substantially widen the uncertainty range of TCRE. For example, it might increase the IPCC's "likely" range of uncertainty from 1.3 °C (IPCC, 2021) to 1.55 °C, representing a 20% increase in uncertainty that linearly propagates into carbon budget calculations. Given this heightened uncertainty and TCRE's sole focus on surface warming, questions arise regarding its practical utility in shaping climate policy. Carbon emissions not only trigger surface warming but also affect various other aspects of the Earth system with varying impacts across different emission pathways."*

1.5 ***Logic around what is expected and what is surprising*** *The paper seems to misrepresent (or its authors misunderstand) what is expected and what is not in terms of the relationship between cumulative CO_2 emissions, atmospheric CO_2 and warming. For example, the difference between pathways in Figure 1c is exactly what you expect. The relationship between cumulative CO_2 emissions and atmospheric CO_2 is pathway-dependent. Similarly, the relationship between atmospheric CO_2 and warming is pathway-dependent. It is the cancellation between these two pathway-dependent relationships that gives rise to the approximately pathway-independent relationship between cumulative CO_2 emissions and warming. If Figure 1c showed warming against cumulative CO_2 emissions then this would be a more unexpected result (but even then, the difference pathways would not be so big relative to overall uncertainty, see also comments about framing below).*

Yes, TCRE is often found to be a constant due to the compensating effects in the system, as we summarize in the manuscript: "*CO_2 emissions translate into a change in global temperature, i. e., TCRE, primarily via three key processes: terrestrial and oceanic CO_2 uptake, the radiative forcing due to atmospheric CO_2, and ocean heat uptake Gregory et al. (2009); Canadell et al. (2021). These processes are governed by complex nonlinear mechanisms, but they appear to balance each other, so that TCRE emerges to be a constant in the coupled climate-carbon cycle system as shown by many modeling exercises Matthews et al. (2009); Gregory et al. (2009); Canadell et al. (2021). However, the TCRE proportionality and its underlying properties may only hold for the prevailing modeling*

paradigm and because of the simplifications made in most modeling exercises, as an analysis of simulations of CMIP5 generation Earth system models has already shown Winkler et al. (2019)."

The commonly accepted explanation posits that the airborne fraction rises as cumulative emissions increase, indicating that sinks become saturated with greater carbon input into the system, following a non-linear, exponential function. However, this non-linearity is offset by the logarithmic function of radiative forcing of atmospheric CO_2 and ocean heat uptake. While this rationale holds true for exponential pathways, where these effects balance each other, we demonstrate that the behavior of the airborne fraction, as a diagnostic of the carbon cycle, can vary depending on emissions pathways. Contrary to expectations, it may not only increase with cumulative emissions but also decrease, contingent upon the pathway trajectory. We further include a detailed decomposition of TCRE to investigate in-depth the compensation processes in our experiments (Figure R2-2).

1.6 *Given this context, there are a few statements which need to be fixed. page 6, line 126-127: "The variation in atmospheric carbon at the end of the emission phase is larger across pathways than models (200 vs. 100 Pg C), which is contrary to expectation". As above, this is exactly what you would expect. If the statement were about the variation in warming then it would be different, but this statement is about the variation in atmospheric* CO_2 *so this variation across pathways is completely in line with expectations.*

Please see our response to the the earlier comment 1.5. We agree with the reviewer that some of the non-linearity in the carbon-cycle response is expected. However, the statement mentioned by the reviewer is referring to the aspect, that these carbon-cycle variations across pathways are larger in one specific model than the variations among the different CMIP6 models for one given pathway. As the modelling community in the carbon cycle and TCRE research quantifies uncertainty mainly in terms of multi-model spread, we were surprised that the changes in atmospheric CO_2 can be stronger among pathways than among different models. This type of uncertainty however does not emerge in the ScenarioMIPs, for example,where the forcing CO_2 time-series are obtained from assessment models.

1.7 *page 6, line 148-150: "Thus, the emergent decay rates are not a model-specific property but depend on the forcing, contrary to previous reporting". I think this understanding of the state-dependence of decay rates is well understood in the literature, indeed it is this state-dependence which motivated the entire design of the FaIR model (Millar et al., 2017). Once again, I don't think this characterisation of previous reporting or differences from current understanding is correct.*

We thank the reviewer for pointing out additional literature. We incorporated the references accordingly and rephrased some sections to account for previous findings:

"*Thus, the emergent decay rates are not a model-specific property but are contingent upon the forcing-induced state of the system, contrary to earlier reporting (Joos et al., 2013), yet consistent with efforts to account for state dependency (Millar et al., 2017).*"

"*Thirdly, another key shortcoming of TCRE research until today is the focus on atmosphere-ocean coupling and the use of simplified modelling approaches therein, such as analytical models (MacDougall, 2016) or so-called ESMs of intermediate complexity (EMICs; e. g., in Herrington and Zickfeld, 2014; Goodwin et al., 2015; Steinacher and Joos, 2016; MacDougall, 2017; Seshadri, 2017). While these methods have advanced our understanding by revealing potential non-linearities and state dependency in TCRE, (e.g., Millar et al., 2017; Nicholls et al., 2020; Avakumović, 2023), the latest studies employing comprehensive ESMs in investigating pathway dependence in fully-coupled emission-driven simulations date back nearly a decade (Zickfeld et al., 2012; Nohara et al., 2013; Krasting et al., 2014).*"

1.8 *page 8, line 167-168: "This time dependency in the terrestrial carbon cycle, hitherto unacknowledged, serves to highlight the dependency of the system on forcing timeline." As above, if this were a discussion of warming then the statement would be correct. As it relates to the state of the carbon cycle, I don't think the statement nor its implications about the state of the literature ("hitherto unacknowledged") are correct.*

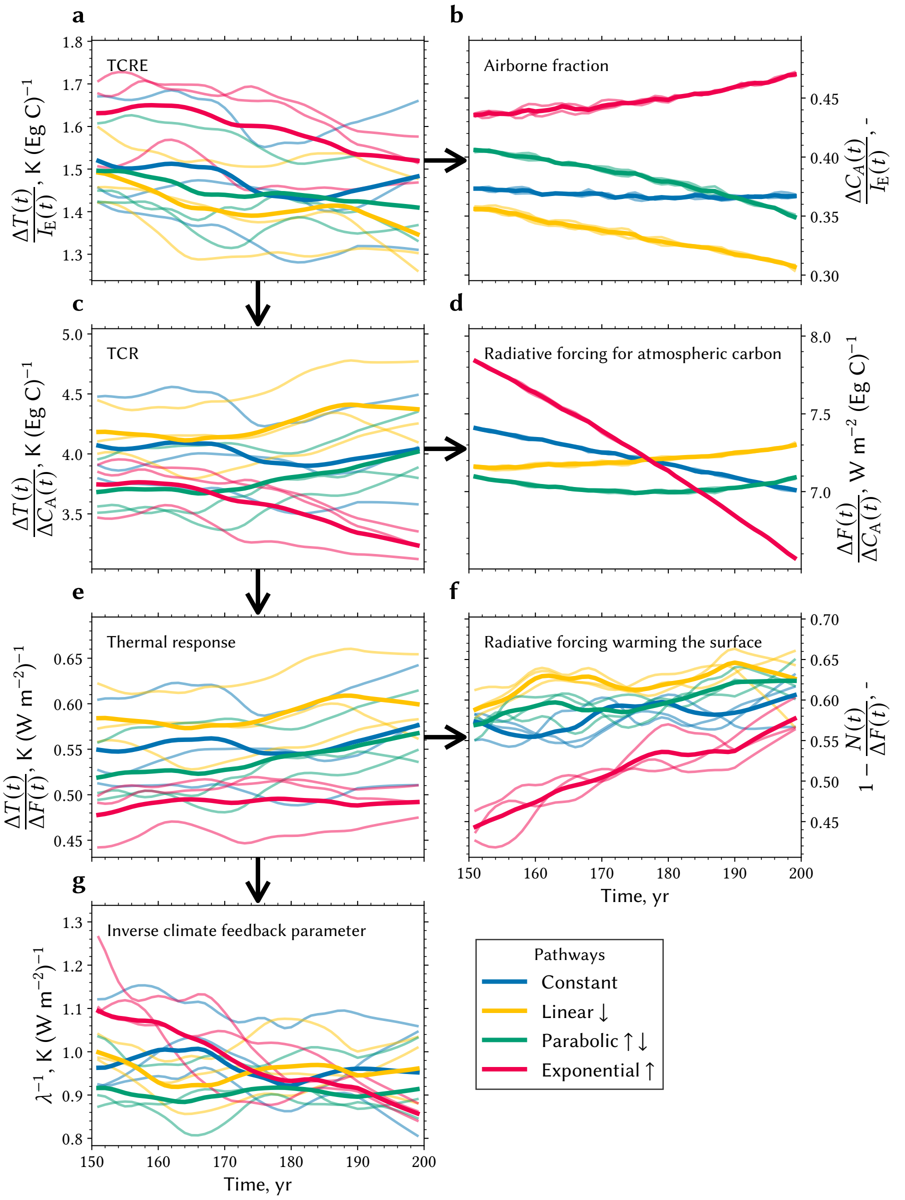

Figure R2- 2: **Decomposition of TCRE into planetary heat uptake, carbon cycling, and physical feedback parameter a** The evolution of TCRE in the four different emission pathways over the last five decades in the 200-year simulations. Each thick line represents the ensemble average of three realizations per experiment smoothed by a low-pass filter (Savitzky-Golay filter). Following the black arrows, TCRE is first decomposed into the airborne fraction of emissions (**b**) and the Transient Climate Response (TCR, **c**). TCR is further decomposed into the radiative forcing of atmospheric carbon (**d**) and the thermal response (**e**), which is again decomposed into the fraction of radiative forcing warming the surface (**f**) and the inverse climate feedback parameter (**g**).

We agree with the reviewer that the carbon-cycle state has been addressed in the literature before. Here, however, we are referring only to the terrestrial carbon cycle and its time dependency. Historically, the terrestrial carbon cycle has been underrepresented in modeling studies of this kind, particularly in terms of vegetation dynamics, a key element for state dependency in the system. We thus argue, that the statement in the manuscript is correct, however, we made it more clear that it refers to terrestrial carbon cycle only.

1.9 ***Framing*** *The paper is written in such a way that, at least to my reading, suggests we need to completely re-think the use of the remaining carbon budget concept. For example, the statement, "Therefore, the emphasis on remaining carbon budget to limit surface warming in accordance with the Paris Agreement requires a critical examination". I don't think such a framing is justifiable.*

We thank the reviewer for the comment. Yes, we agree and removed the sentence. We adapted the framing such that it focuses more on the aspect of additional uncertainty in TCRE and carbon budgets associated to our findings:

"These shortcomings may introduce previously unconsidered uncertainties into TCRE and the resulting remaining carbon budget (Matthews et al., 2009; Zickfeld et al., 2009, 2012; MacDougall, 2016; Williams et al., 2020; IPCC, 2021), potentially affecting efforts to achieve surface warming targets outlined in the Paris Agreement (Rogelj et al., 2016)."

"While these non-linearities do not entirely invalidate TCRE, they do introduce additional uncertainty. If the model-specific range of 0.25 °C for TCRE, as quantified in this study, were to apply in all models in this fully-coupled setting, it could substantially widen the uncertainty range of TCRE. For example, it might increase the IPCC's "likely" range of uncertainty from 1.3 °C (IPCC, 2021) to 1.55 °C, representing a 20% increase in uncertainty that linearly propagates into carbon budget calculations. Given this heightened uncertainty and TCRE's sole focus on surface warming, questions arise regarding its practical utility in shaping climate policy."

1.10 *The remaining carbon budget is a key concept in climate science that has been heavily examined for over a decade. It is probably true that its use often needs to be more clearly accompanied with the caveat that it is a first-order approximation, and that more sophisticated tools are required for more precise estimates. However, using a linear TCRE is often still appropriate because of the wide uncertainties associated with the TCRE. For example, in the latest IPCC report the likely (17-83%) range for the TCRE was 1.0 - 2.3 K / TtC. Against an uncertainty of this size, a +/- 20% deviation away from a constant TCRE is irrelevant for most use cases, such as for the IPCC's reporting of remaining carbon budgets. The authors must do a better job of capturing the size of the deviation they are discussing compared to wider uncertainties in the TCRE if they are to frame this paper appropriately and to highlight the circumstances in which their analysis requires a re-think and the circumstances in which the corrections they are discussing are small compared to other uncertainties.*

We thank the reviewer for this insightful comment. We agree that TCRE linearity is still an appropriate first-order approximation in most cases. The crucial point here is that IPCC's estimate on TCRE uncertainty is based on multi-model statistics, so it reflects the model spread. We report deviations in TCRE within a single model. Accordingly, for the sake of the argument, if all models accounted for a terrestrial carbon state, all models would be subject to a deviation of the order of 0.25 K, which would propagate into the multi-model informed uncertainty. Please see also our response to comment 1.9 with respect to how we changed the framing in the revised manuscript.

1.11 ***Precision*** *Related to the above point, it would be easier for the reader to understand the size of the deviations being discussed if the authors removed all vague quantifications, such as "roughly constant" in the abstract. A similarly vague quantification is used on page 10, line 206-207: "However, at the same state of the system, the sink efficiency is the same irrespective of the pathway property (Figure 4c)." In the figure, all the dots are clearly not the same so it would be easier if these statements were quantified, rather than only described qualitatively. This would also remove the need for the authors to judge what is the same and what is different (a choice I can't really understand as the dots in Figure 4c seem to be as spread as in other figures yet they are described as the same whereas a similar spread is described as a difference elsewhere).*

We appreciate the reviewer's critical comment. While we strive to incorporate quantifications where appropriate, there are instances, such as the one mentioned, where we discuss relationships qualitatively to emphasize key points, such as the convergence of sink efficiency. Nevertheless, we have revised these passages to enhance clarity and adopt alternative wording to better convey our intentions.

2 Minor Concerns

1.12 ***Acknowledgement of other studies*** *The authors frame this paper in a way that suggests that everyone is assuming the TCRE is perfectly linear in all cases and no-one has ever considered otherwise. For example, the sentence ending in, "which heretofore have been mainly overlooked", in the abstract. I don't think this is a fair framing. There have been studies which consider non-linearities in the TCRE and their impact (see, e.g. Nicholls et al., 2020; Avakumović. 2023 and references therein). These studies should be acknowledged and considered. (I assume the authors will disagree with the models that sit underneath these studies, but that is besides the point, which is that people have thought about path- and state-dependency in the TCRE already and presenting the literature as if no-one has is misleading.)*

We thank the reviewer for pointing out these studies. We studied and implemented new a body of literature, including Nichols et al. (2020); Avakumović (2023) as well as Sanderson et al. (2023); Jenkins et al. (2022); Koven et al. (2023); Millar et al. (2017).

1.13 ***Reproducibility*** *As a reviewer, part of my job is to check the reproducibility of the results. I acknowledge that this is rarely done in climate science for a whole range of reasons (hence why this is a minor concern rather than major), but I think it is worth trying to do this better here. My key concerns are that, as a reviewer, I cannot: inspect or run any of the code used for downloading and processing the CMIP results*

Certainly, we acknowledge and agree with this comment. Reproducibility is a pivotal aspect, and it's an ongoing challenge in climate science. We address this concern in detail below.

1.14 *download or run any of the processing/plotting code (if I could, maybe my complaint about missing figures could have been dealt with by me just making the figure myself)*

Many again thanks for this point. We published the processing and plotting scripts and the key data underlying the plots in a zenodo repository: https://zenodo.org/records/10905388.

1.15 *access any of the model output required to make the figures/reproduce the analysis without asking the author: I strongly encourage the authors to find a way to make the output data publicly available, there are solutions for dealing with large data files so I don't think that is a fair justification for not releasing the data (and if concerns about being scooped are part of the reason, a suitable license for data access should deal with this)*

Certainly, we agree with the reviewer that ideally, all model output, including temporal 3-dimensional fields of ocean and atmosphere, as well as temporal 2-dimensional fields of surface variables of the entire ensemble, should be made publicly available. However, hosting such a large amount of model output on a public server presents logistical challenges. Additionally, resource management is a consideration; hosting large amounts of data that may only be downloaded a few times is not cost-effective. We have included the key variables and key processed time series in a publicly available repository on Zenodo. You can access the data repository on Zenodo through the provided link: https://zenodo.org/records/10905388

1.16 *inspect any of the scripts used to configure and run MPI-ESM. Having read through MPI-ESM's RECIPE file, this is clearly an involved process. In an ideal world, the ability to re-run the model would be there. However, given MPI-ESM's run setup is specific to MPI's computing environment, that doesn't seem to be possible (which is the standard in climate science unfortunately). Given this, at a minimum the run scripts and any custom inputs should be archived next to this paper to allow for at least a non-zero chance of reproducibility.*

Again, we appreciate the reviewer's important comment regarding reproducibility. It is indeed crucial to make efforts to enhance reproducibility in climate science. Alongside the analysis

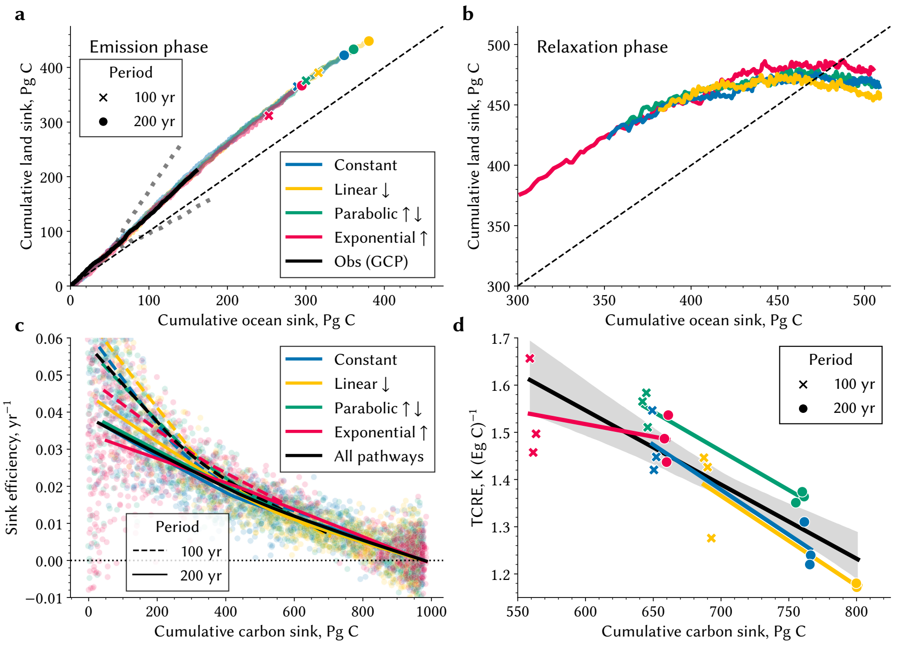

Figure R2- 3: **State of the carbon system, sink efficiency, and TCRE across the pathways.** **a** Cumulative land and ocean sinks are juxtaposed across all pathways and both simulation periods. The markers denote the final state at the end of the emission phase of 1.2 Eg C. The dashed line indicates equal magnitude of cumulative land and ocean sinks. The black line refers to the average relationship inferred from GCP estimates, where the gray dots follow plus/minus the joint uncertainty for both cumulative land and ocean sink. **b** As in a, but for the relaxation phase. **c** Sink efficiency is plotted as function of the cumulative carbon sink for both emission and relaxation phase. The lines represent a nonparametric LOWESS fit, with the solid (dashed) line referring to the 200-(100-)year simulations in the different pathways indicated by different colors. The black lines indicate the relationship when all pathways are fitted at once. **d** TCRE is plotted as function of the cumulative carbon sink, *i. e.,* the state of the carbon system. The colored lines represent a linear fit for the four different pathways. The black lines indicate the relationship when all pathways and both periods are fitted linearly at once. The grey shading reflects uncertainty based on an 95% confidence interval estimated using bootstrapping.

scripts, we have published the MPI-ESM1.2 run scripts and the scripts responsible for generating the idealized CO_2 emissions files. These scripts are aimed at facilitating generalization so that they can be modified to produce forcing files for other Earth system models (ESMs). You can access these scripts in the provided Zenodo repository link.

3 Figures

1.17 *Very nice and clear, thank you. One small question: Is it possible to add uncertainty bars/shading onto the GCP estimate in Fig S7 and Fig 4a?*

Many thanks for the appreciation and the question. Yes, this is possible and a very good suggestion. We updated the figure by plotting not only the multi-model means, but also the multi-model spread in terms of the standard deviation as provided by the GCP. Figure 3

4 Technical corrections

2.1 *page 3, line 29: 'time sequence and duration' → 'timing'*

We agree with the reviewer and replaced 'time sequence and duration' by 'timing' in the revised manuscript.

2.2 page 3, line 31: delete 'likely' (unless there is some justification, in which case add it)

We removed the word 'likely' in the revised manuscript.

2.3 page 5, line 92: 'into the' → 'into'

We thank the reviewer for spotting the mistake. We corrected the sentence.

2.4 page 7, line 156-157: "These model intercomparison exercises lack dynamics arising from an interactive carbon cycle as these are CO_2 concentration driven simulations only" That's only true if you don't have a cumulative CO_2 emissions axis in your analysis/plots. If you have that axis, there has to be some carbon cycle influence in there no (because the carbon cycle determines uptake, so you will get variations even though it is a concentration-driven experiment)?

We do not fully understand the reviewer's comment. CO_2 concentration-driven simulations do not allow for interactions between the different compartments of the carbon cycle, *i.e.*, the land and ocean carbon cycle feedback is locked, since the atmospheric CO_2 is prescribed. This means that changes in sinks and sources characteristics in each compartment have no effect on the atmospheric CO_2. So, there is no carbon-cycle influence on the atmospheric CO_2 concentration, but there is some biogeophysical effects like changes in albedo, evapotranspiration, surface roughness, etc.

2.5 page 15, line 294: "negligible" → "negligibly"

Many thanks for spotting the typo.

2.6 page 16, line 317-318: "resulting in strong vertical gradients in the mixing ratio of CO_2, causing the model to crash", sounds painful, well done for working out how to handle that

Many thanks for the appreciation!

2.7 page 31, line 602-603: reference incomplete, current text says "point to methods"

Thanks for spotting the incomplete reference. We corrected the reference in the revised manuscript.

2.8 page 32, line 618: 'but for' → 'but for the'

Many thanks for the pointing this out. We corrected the mistake.

References

Avakumović, V. (2023). Carbon budget Concept, its Deviation and the Equation: Climate Economics Perspective. *Earth System Dynamics Discussions*, pages 1–28.

Canadell, J. G., Monteiro, P. M., Costa, M. H., da Cunha, L. C., Cox, P. M., Eliseev, A. V., Henson, S., Ishii, M., Jaccard, S., Koven, C., Lohila, A., Patra, P. K., Piao, S., Syampungani, S., Zaehle, S., Zickfeld, K., Alexandrov, G. A., Bala, G., Bopp, L., Boysen, L., Cao, L., Chandra, N., Ciais, P., Denisov, S. N., Dentener, F. J., Douville, H., Fay, A., Forster, P., Fox-Kemper, B., Friedlingstein, P., Fu, W., Fuss, S., Garçon, V., Gier, B., Gillett, N. P., Gregor, L., Haustein, K., Haverd, V., He, J., Hewitt, H. T., Hoffman, F. M., Ilyina, T., Jackson, R., Jones, C., Keller, D. P., Kwiatkowski, L., Lamboll, R. D., Lan, X., Laufkötter, C., Quéré, C. L., Lenton, A., Lewis, J., Liddicoat, S., Lorenzoni, L., Lovenduski, N., MacDougall, A. H., Mathesius, S., Matthews, D. H., Meinshausen, M., Mokhov, I. I., Naik, V., Nicholls, Z. R. J., Nurhati, I. S., O'Sullivan, M., Peters, G., Pongratz, J., Poulter, B., Sallée, J.-B., Saunois, M., Schuur, E. A., I.Seneviratne, S., Stavert, A., Suntharalingam, P., Tachiiri, K., Terhaar, J., Thompson, R., Tian, H., Turnbull, J., Vicente-Serrano, S. M., Wang, X., Wanninkhof, R., Williamson, P., Brovkin, V., Feely, R. A., and Lebehot, A. D. (2021). Global Carbon and other Biogeochemical Cycles and Feedbacks. In *Climate Change 2021: The Physical Science Basis. Contribution of Working Group I to the Sixth Assessment Report of the Intergovernmental Panel on Climate Change*. Cambridge University Press, Cambridge, United Kingdom and New York, NY, USA.

Goodwin, P., Williams, R. G., and Ridgwell, A. (2015). Sensitivity of climate to cumulative carbon emissions due to compensation of ocean heat and carbon uptake. *Nature Geoscience*, 8(1):29–34.

Gregory, J. M., Jones, C. D., Cadule, P., and Friedlingstein, P. (2009). Quantifying Carbon Cycle Feedbacks. *Journal of Climate*, 22(19):5232–5250.

Herrington, T. and Zickfeld, K. (2014). Path independence of climate and carbon cycle response over a broad range of cumulative carbon emissions. *Earth System Dynamics*, 5(2):409–422.

IPCC (2021). *Climate Change 2021: The Physical Science Basis. Contribution of Working Group I to the Sixth Assessment Report of the Intergovernmental Panel on Climate Change.* Cambridge University Press.

Jenkins, S., Sanderson, B., Peters, G., Frölicher, T. L., Friedlingstein, P., and Allen, M. (2022). The Multi-Decadal Response to Net Zero CO2 Emissions and Implications for Emissions Policy. *Geophysical Research Letters*, 49(23):e2022GL101047.

Joos, F., Roth, R., Fuglestvedt, J. S., Peters, G. P., Enting, I. G., von Bloh, W., Brovkin, V., Burke, E. J., Eby, M., Edwards, N. R., Friedrich, T., Frölicher, T. L., Halloran, P. R., Holden, P. B., Jones, C., Kleinen, T., Mackenzie, F. T., Matsumoto, K., Meinshausen, M., Plattner, G.-K., Reisinger, A., Segschneider, J., Shaffer, G., Steinacher, M., Strassmann, K., Tanaka, K., Timmermann, A., and Weaver, A. J. (2013). Carbon dioxide and climate impulse response functions for the computation of greenhouse gas metrics: A multi-model analysis. *Atmospheric Chemistry and Physics*, 13(5):2793–2825.

Koven, C. D., Sanderson, B. M., and Swann, A. L. S. (2023). Much of zero emissions commitment occurs before reaching net zero emissions. *Environmental Research Letters*, 18(1):014017.

Krasting, J. P., Dunne, J. P., Shevliakova, E., and Stouffer, R. J. (2014). Trajectory sensitivity of the transient climate response to cumulative carbon emissions. *Geophysical Research Letters*, 41(7):2520–2527.

MacDougall, A. H. (2016). The Transient Response to Cumulative CO_2 Emissions: A Review. *Current Climate Change Reports*, 2(1):39–47.

MacDougall, A. H. (2017). The oceanic origin of path-independent carbon budgets. *Scientific Reports*, 7(1):10373.

Matthews, H. D., Gillett, N. P., Stott, P. A., and Zickfeld, K. (2009). The proportionality of global warming to cumulative carbon emissions. *Nature*, 459(7248):829–832.

Millar, R. J., Nicholls, Z. R., Friedlingstein, P., and Allen, M. R. (2017). A modified impulse-response representation of the global near-surface air temperature and atmospheric concentration response to carbon dioxide emissions. *Atmospheric Chemistry and Physics*, 17(11):7213–7228.

Nicholls, Z. R. J., Gieseke, R., Lewis, J., Nauels, A., and Meinshausen, M. (2020). Implications of non-linearities between cumulative CO2 emissions and CO2-induced warming for assessing the remaining carbon budget. *Environmental Research Letters*, 15(7):074017.

Nichols, R. E., Subrahmanyam, B., and Arguez, A. (2020). Recent Variability in the Arctic Ocean and Subarctic Seas. *Remote Sensing in Earth Systems Sciences*, 3(1):44–54.

Nohara, D., Yoshida, Y., Misumi, K., and Ohba, M. (2013). Dependency of climate change and carbon cycle on CO2 emission pathways. *Environmental Research Letters*, 8(1):014047.

Rogelj, J., den Elzen, M., Höhne, N., Fransen, T., Fekete, H., Winkler, H., Schaeffer, R., Sha, F., Riahi, K., and Meinshausen, M. (2016). Paris Agreement climate proposals need a boost to keep warming well below 2 °C. *Nature*, 534(7609):631–639.

Sanderson, B. M., Booth, B. B. B., Dunne, J., Eyring, V., Fisher, R. A., Friedlingstein, P., Gidden, M. J., Hajima, T., Jones, C. D., Jones, C., King, A., Koven, C. D., Lawrence, D. M., Lowe, J., Mengis, N., Peters, G. P., Rogelj, J., Smith, C., Snyder, A. C., Simpson, I. R., Swann, A. L. S., Tebaldi, C., Ilyina, T., Schleussner, C.-F., Seferian, R., Samset, B. H., van Vuuren, D., and Zaehle, S. (2023). The need for carbon emissions-driven climate projections in CMIP7. *EGUsphere*, pages 1–51.

Seshadri, A. K. (2017). Origin of path independence between cumulative CO_2 emissions and global warming. *Climate Dynamics*, 49(9):3383–3401.

Steinacher, M. and Joos, F. (2016). Transient Earth system responses to cumulative carbon dioxide emissions: Linearities, uncertainties, and probabilities in an observation-constrained model ensemble. *Biogeosciences*, 13(4):1071–1103.

Williams, R. G., Ceppi, P., and Katavouta, A. (2020). Controls of the transient climate response to emissions by physical feedbacks, heat uptake and carbon cycling. *Environmental Research Letters*, 15(9):0940c1.

Winkler, A. J., Myneni, R. B., and Brovkin, V. (2019). Investigating the applicability of emergent constraints. *Earth System Dynamics*, 10(3):501–523.

Zickfeld, K., Arora, V. K., and Gillett, N. P. (2012). Is the climate response to CO_2 emissions path dependent? *Geophysical Research Letters*, 39(5).

Zickfeld, K., Eby, M., Matthews, H. D., and Weaver, A. J. (2009). Setting cumulative emissions targets to reduce the risk of dangerous climate change. *Proceedings of the National Academy of Sciences*, 106(38):16129–16134.
