# Supplementary material for: Carbon system state determines warming potential of emissions
Source: PLoS One. 2024 Aug 1;19(8):e0306128. doi: 10.1371/journal.pone.0306128 (PMC11293723; doi:10.1371/journal.pone.0306128)
Supplement: Attachment — Submitted filename: response-to-ref-02.pdf [file pone.0306128.s013.pdf]

Authors' Response to Referee 2 (PONE-D-23-32708)

May 31, 2024

For what it's worth, I still think the authors need to do a better job of explaining how their conclusion fits with current knowledge. I don't think a paper based on a single model that says, "Throw away everything you thought you knew about TCRE being pathway independent", without explaining exactly how they come to such a strong conclusion, is justifiable. If they can say something like, "The TCRE appears pathway independent because all our simulations basically use the same sort of carbon cycle state", (or some other explanation of why it has appeared that the TCRE is pathway-independent for so long and no-one has noticed that it isn't), that would be great. Alternately, they might just say, "All the effects we're looking at here lead to deviations of 10% from a pathway-independent TCRE", in which case their work is completely in line with previous work e.g. https://esd.copernicus.org/preprints/esd-2023-7/ and https://iopscience.iop.org/article/10.1088/1748-9326/ab83af

We thank the reviewer for reviewing the revised version of our manuscript and for the critical comment. Our study does not conclude "throw away everything you thought you knew about TCRE being pathway independent", but rather we say that the state of the system, and in particular the state of the carbon cycle, must be taken into account when estimating the warming potential of carbon emissions. Yes, the main results are based on a single model, but there is only a very limited number of models that provide the comprehensive representation of terrestrial carbon dynamics, particularly vegetation dynamics, required for such a study. We hope that this study will serve as an incentive for efforts to better represent vegetation dynamics in other coupled Earth system models. We have already included the references suggested by the reviewer. However, we agree with the reviewer that some points need to be communicated more clearly. The abstract now contains the following statement:

While this study does not fundamentally invalidate the established TCRE concept, it does uncover additional uncertainties tied to the carbon system state. Thus, efforts to better understand this state dependency with observations and refined models are needed to accurately project the impact of future emissions.

And the conclusions now contain a more precise statement on the implications of the study for the TCRE concept:

While these non-linearities do not invalidate TCRE, they do introduce additional uncertainty. If the model-specific range of 0.25 °C for TCRE, as quantified in this study, were to apply in all models in this fully-coupled setting, it could substantially widen the uncertainty range of TCRE. For example, it might increase the IPCC's "likely" range of uncertainty from 1.3 °C **?** *to 1.55 °C, representing a 20% increase in uncertainty that linearly propagates into carbon budget calculations.*
